# Copper Accumulation in the Lips of Brass Players: Case Report of a Rare Phenomenon

**DOI:** 10.3390/dj10110203

**Published:** 2022-10-27

**Authors:** Zoltán Baráth, Nóra Heltai, Éva Kereszty, Ildikó Kiss, Márió Gajdács, Nándor Tamás Práger, Krisztina Kárpáti, Danica Matusovits

**Affiliations:** 1 Department of Prosthodontics, Faculty of Dentistry, University of Szeged, Tisza Lajos körút 64-66, 6720 Szeged, Hungary; 2 Department of Forensic Medicine, Albert Szent-Györgyi Health Centre and School of Medicine, University of Szeged, Kossuth L. sgt. 40, 6724 Szeged, Hungary; 3 Affidea Hungary Ltd., Semmelweis u. 6, 6725 Szeged, Hungary; 4 Department of Oral Biology and Experimental Dental Research, Faculty of Dentistry, University of Szeged, Tisza Lajos körút 64-66, 6720 Szeged, Hungary; 5 Private Dental Clinic, Arany János u. 3, 6900 Makó, Hungary; 6 Department of Orthodontics and Pediatric Dentistry, Faculty of Dentistry, University of Szeged, Tisza Lajos körút 62-64, 6720 Szeged, Hungary

**Keywords:** copper, heavy metal, occupational health, occupational exposure, oral health, magnetic resonance imaging, MRI artefact, brass player, mouthpiece

## Abstract

Work-related exposures affecting oral health are important factors of morbidity and decreased quality of life, which may emerge from numerous physical, chemical, or mental occupational exposures. Copper (Cu) is an important trace element, however, it may also cause allergies, depose and accumulate in the body, leading to acute and chronic toxicity. In the present report, we describe a rare phenomenon found during the examination of two professional brass players, after incidentally noting an artefact during magnetic resonance imaging (MRI) scans, which were performed to monitor bone healing after bone augmentation procedures in an unrelated clinical study. During a detailed workup of patient characteristics, data on medical history, lifestyle, professional habits related to playing the instrument, and oral health status were collected. Overall, both patients presented with similar histories, and the differences from the context of this study were not relevant; however, both brass players were using an uncoated Cu mouthpiece for over 15 years. Based on the imaging findings (a shadow in the area of the lips on the MRI images) and the organoleptic evaluation of the lips and mucosa of the individuals (temporary faint green discoloration), it is most likely that the brass players were affected by oxidized Cu accumulation in the lip. In contrast to several professions, musicians are usually not required by law to attend obligatory occupational health check-ups, which may facilitate the occurrence of such exposures in musicians. Clinicians should be on the lookout for brass players involved in the profession for a long time, who may have used the mouthpieces for an extended period of time, in addition to musicians affected by Wilson’s disease. In patients affected by this phenomenon, diagnostics of oral cancer and prosthodontic procedures may be cumbersome, due to the detrimental impact on the utility of MRI imaging from artefact-formation and scattering.

## 1. Introduction

Chronic occupational exposures and accidents affecting an individual’s health are considered an important factor of morbidity, decreased quality of life, and mortality worldwide [1]. Occupational diseases—per the World Health Organization (WHO) and International Labour Organization (ILO) definition—result from exposure to relevant risk factors arising from work activities [2]. Such work-related disorders may stem from various exposures, such as mechanical (e.g., noise), chemical (e.g., exposure to carcinogens, asthmagens, and polluted air), ergonomic risk factors, long working hours, and psychological factors (e.g., chronic stress, allostatic load) [3]. While many studies have reported estimates on the mortality associated with occupational diseases, reliable morbidity data on the topic is scarce; based on WHO/ILO reports, around 1.9 million people have died worldwide due to work-related exposures or injuries, out of which, over 80% of deaths have been caused by non-communicable diseases [4].

Work-related exposures affecting the oral cavity may be considered representative examples leading to such non-communicable diseases [5]. It is now well-known that teeth and the surrounding soft tissues may be affected by numerous physical, chemical, or mental occupational factors. Employees may develop—besides teeth and gingival changes—temporomandibular joint (TMJ) disorders, muscle dysfunctions, chronic pain, and psychological distress [6]. Cement and sand workers, grinders, stone cutters, and miners may be considered are noteworthy examples, as the abrasive dust may lead to the staining of the teeth, gingival pigmentation, development of calculi, generalized abrasion, gingivostomatitis and hemorrhage [7]. A high prevalence of dental caries may be noted among the workers of sugar refineries, bakers, and pastry cooks due to sugar dust [8]. Similarly, in individuals exposed to various acidic substances (e.g., wine-makers, chemists) demineralization caused erosion is also noteworthy [9]. 

Abrasion (or mechanical wear) of the dentition has been associated with several occupations (such as shoe-makers, upholsterers, glass blowers, dress designers, and seamstresses), resulting from prolonged interaction with objects (other than tooth-tooth contact), like nails, tacks, needles and glass tubes [10]. Due to the psychological effects and high-stakes environment in certain occupations, people may also develop bruxism (e.g., athletes, and paramedics). Likewise, masseter muscle hypertrophy has also been described in weight-lifters or “strongmen” [11]. In addition to hypersensitivity reactions, toxic exposure to metals (e.g., nickel [Ni], mercury [Hg], iron [Fe], chromium [Cr], lead [Pb], manganese [Mn], zinc [Zn], silver [Ag], gold [Au], and copper [Cu]) through dust or direct contact may also lead to a variety of disease presentations and symptoms, both in the oral cavity and for systemic health [12]. 

As previously mentioned, morbidity data associated with occupational exposures are scarce, which is especially true for work-related oral disorders; most of the information has been derived from health insurance claims data, and from surveillance data of larger corporations or the public sector, providing health insurance covering oral-health related occupational medicine services [13]. It has also been described that the overall oral health status of individuals may be considerably better, if they are employed by companies offering screening for oral diseases in their healthcare plans, compared to entrepreneurs or musicians/artists, who are often forced to opt for private health insurance [14]. Musicians are also at risk for several occupational exposures, including ergonomic stress (holding the instrument in a specific position, leading to musculoskeletal diseases) and mechanical factors (loud, constant, and repetitive sound, leading to a hearing loss) [15]. Furthermore, the mouthpieces (often called embouchures) of brass and woodwind instruments may also cause changes in the oral cavity. 

Brass instruments may differ in their chemical composition, with a specific combination of materials (usually a Cu-Zn alloy in different proportions), while folk instruments are mainly composed of natural materials, i.e., ceramics or wood [16]. The most commonly reported orofacial changes of brass players are malocclusion, xerostomia, dry and painful lips, TMJ disorders, bruxism, and contact dermatitis/allergies [17]. Numerous articles have been published regarding the exposures and risks to oral health in musicians [16,18,19,20,21], however, no previous study has reported the accumulation of metal particles in the soft tissues of the lips, associated with playing brass instruments. In the present report, we describe a rare phenomenon found during the examination of two professional brass players, after incidentally noting an artefact during magnetic resonance imaging (MRI) scans, which was most likely related to this phenomenon.

## 2. Materials and Methods

The study has been initiated after an accidental finding on a patient’s MRI scans, which were performed to monitor bone healing after bone augmentation procedures in an unrelated clinical study: a round-shaped shadow was spotted in the lips’ area of the scan. The first patient’s incidental finding on the MRI scan was seen as an artefact manifestation of the Co-Cr metal ceramic bridge in the frontal maxillary region. As a part of the study, two professional brass players were examined in detail, who both have been using an uncoated Cu mouthpiece for over 15 years. 

Following the patients’ informed written consent, a detailed workup of patient history and anamnesis was performed. Firstly, general patient history was evaluated by uniform questions about health status, underlying diseases, medication history, and known allergies. Following that, the lifestyle habits of the patients were also assessed, i.e., foods and drinks consumed, smoking, and alcohol use. In addition, a detailed dental anamnesis was taken, including the routine assessment of the general oral health status, caries experience, periodontal status, presence of fillings, dental prostheses, and overall oral hygiene habits. Lastly, professional habits were surveyed, like the type of mouthpiece they used, instrument use and instrument cleaning habits, or any rituals preceding/during/after playing the instrument. 

The thorough history-taking aimed to exclude other potential factors that could have caused the presence of metal particles in the lips. Lastly, during MRI scanning, the following, adjusted directions and measurements were used to decrease the influence of the artefact and the subsequent scattering, and to produce more relevant scans: T2 ax-20.83, T2 ax-41.67, T1 cor-20.83, T1 cor-41.67, T1 sag-25.00, T1 sag-50.00, T1 ax-20.83, T1 ax-41.67 Bandwith measurements.

## 3. Results (Case Report)

Differences noted in the medical history of the patients are summarized in Table 1. Both patients were male, in a similar age bracket (63 and 61 years, respectively), who played brass professionally with uncoated mouthpieces for approximately the same time (~15 years). The nutritional habits of both patients were unremarkable, neither of them had any allergies and none of them reported any behavioral or consumptive rituals (i.e., no specific food or drink was consumed before/during/after practice) associated with playing the instrument. The patients rarely consumed alcohol, and both of them were smokers at different intensities. While the oral hygiene habits of both brass players were unremarkable, both of them had an impacted oral health status. Overall, both patients presented with similar histories, and the differences from the context of this study were not relevant. Both patients reported noting temporary faint green discoloration of the lips following long practices in the past; the remnants of metal particle (presumably Cu) accumulation could be faintly seen upon examination of the lips and oral mucosa of these professional brass players.

**Figure 1:**
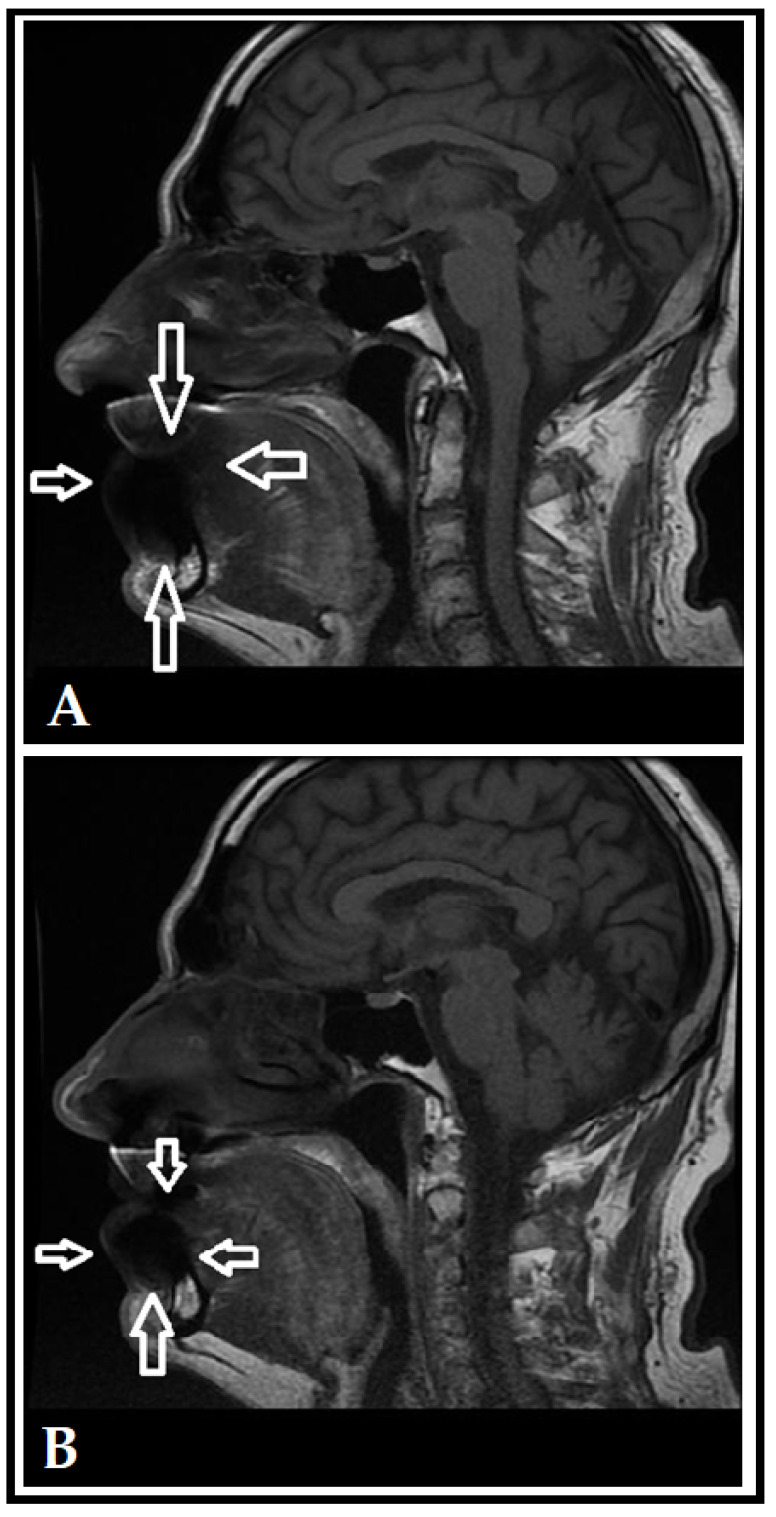
Unadjusted (**A**) and adjusted (**B**) MRI scans for Patient 1 (T1 sag). The shadow in the area of the lips (denoted by white arrows) indicates the imaging artefact caused by Cu accumulation in the lips, which is less pronounced after using adjusted directions and measurements.

**Figure 2:**
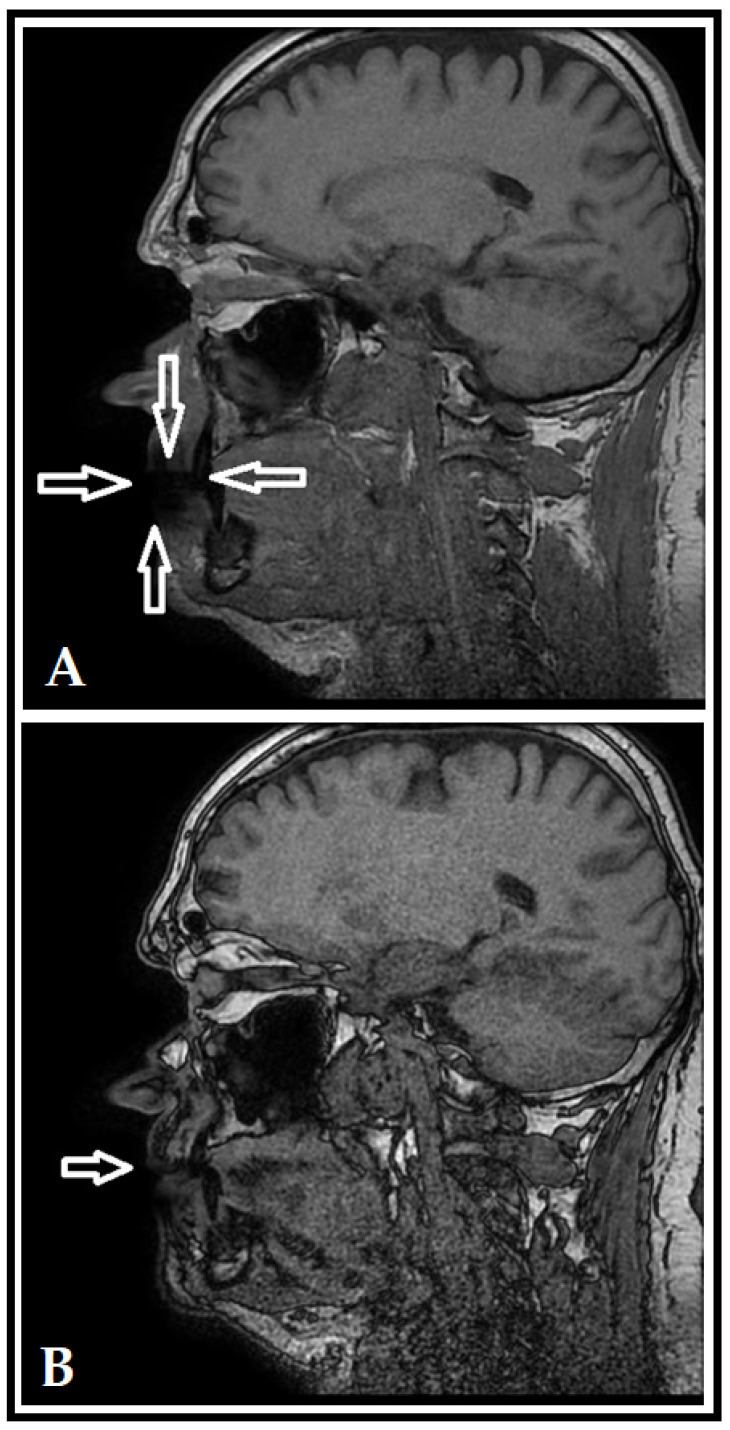
Unadjusted (**A**) and adjusted (**B**) MRI scans for Patient 2 (T1 sag). The shadow in the area of the lips (denoted by white arrows) indicates the imaging artefact caused by Cu accumulation in the lips, which is less pronounced after using adjusted directions and measurements.

On the MRI scans (see Figure 1 and Figure 2) a shadow may be spotted in the area of the lips, referring to Cu accumulation; on the unadjusted images (Figure 1A and Figure 2A), the extent of considerable artefact-formation and scattering may be observed, while the scans with adjusted directions and measurements (Figure 1B and Figure 2B) showed that the influence of the artefact could be decreased. After identifying the accumulation of Cu in these patients based on clinical and imaging findings, the two brass players were referred to a consultation with an internal medicine specialist and to the Department of Occupational Medicine to discuss detoxification interventions and potential preventive measures, respectively. Overall, no pharmacological detoxification measures were initiated, on the other hand, the patients were instructed to switch to a coated mouthpiece; however, both brass players have discontinued using uncoated Cu mouthpieces years before the incidental findings on the MRI scans were noted. Since the identification of the artefact, both patients attended their yearly routine dental check-ups, which were unremarkable. 

## 4. Discussion

In this report, our aim was to present and investigate a rare phenomenon found accidentally on a patient’s MRI scan, which has led to the examination of two professional brass players, who have been using an uncoated copper mouthpiece for more than 15 years, i.e., the lips of these musicians have been in persistent contact with the metal when playing. Other than their extensive work experience, the mouthpiece they used, and smoking, no remarkable anamnestic data was common among the two patients that would explain the phenomenon serendipitously discovered on the MRI scans. Based on the imaging findings and the organoleptic evaluation of the lips and mucosa of the individuals, it is most likely that the brass players were affected by oxidized copper accumulation in the lip, distorting the diagnostic capacity of MRI. 

Cu is a trace element, which is present in our body under physiological conditions and is essential for the function of various enzymes involved in electron transport and oxidation processes [22]. Nonetheless, similar to other heavy metals, Cu may also cause allergies, depose and accumulate in the body, leading to acute and chronic toxicity [23]. In addition, Cu poisoning has also been described as a possible occupational exposure [24]. Symptoms of chronic Cu exposure include abdominal pain, liver failure, and anemia [25]; however, neither these symptoms nor Cu-hypersensitivity were noted in our patients. In various metabolic disorders associated with Cu-accumulation—like Wilson’s disease, an autosomal recessive genetic disorder—it has been described that metal accumulation has oral (gingival) predilection areas, which was also not seen in our subjects [25]. 

Instances of Cu-related occupational exposure are rare findings, which are very uncommonly reported: Donoghue et al. published a similar case report in 1995, where superficial Cu staining was found on the cervical margin of a patient’s teeth, who worked at a brass foundry [26]. Here, inhalation of Cu dust has led to a greenish stain on the teeth of the subject; this mechanism is also possible among musicians who use brass instruments, as dissolution and deposition of various metals may occur due to the close contact with the mucosa and the continuous soaking and maceration of the metal surfaces. The deposition of Cu may also give rise to oral galvanism (or galvanic shock), i.e., the occurrence of electric currents between the metals present in the oral cavity (e.g., from amalgam fillings, restorations) and the electrolytes in the saliva [27,28]. The extent of oral galvanism may be influenced by the restoration’s age, the total surface area of the galvanic couple, and the extent of the contamination [29]. If unresolved, the potential difference present in the oral cavity can lead to the formation of leukoplakia on the oral mucosa, which may be a precursor for the development of malignancy [30]. Nevertheless, this phenomenon may be prevented with the use of coated mouthpieces for brass instruments.

Cu—in low pH conditions—is oxidized by oxygen in the air, which leads to the formation of the green layer deposit on surfaces [31]. Following longer practices with the instruments, our subjects reported experiencing green discoloration temporarily in the areas coming to contact with the uncoated mouthpiece. Chronic smoking (which was characteristic for both patients, and has been reported to decrease the pH of both stimulated and unstimulated saliva [32]), and other acidic materials introduced through nutrition may establish the conditions locally, so the oxidation and accumulation of Cu (in oxide, carbonate and acetate forms) could in the soft tissue of the lip, without leading to any acute or chronic systemic toxicity [33]. Preceding our imaging findings, the patients have stopped using uncoated Cu mouthpieces for several years, therefore our assumption was that the Cu levels in their blood would not be elevated. A lip biopsy could have had diagnostic value, however, this invasive method could have detrimental effects on the musicians’ ability to play the instrument, therefore, this option was disregarded. In contrast to several professions, musicians are usually not required by law to attend obligatory occupational health checks, in addition, they often resort to utilizing private healthcare as needed. Being outside the “scope” of continuous healthcare screening may facilitate the occurrence of such exposures in musicians. The affected patients were identified based on an artefact seen during MRI imaging. From an imaging aspect, the phenomenon of Cu accumulation distorts the regular MRI diagnostic value; this might lead to a critical issue in limiting oncogenic diagnostics. Such artefacts may lead to difficulties in determining the localization and borders of squamous cell carcinomas of the upper lip, which are among the most commonly diagnosed oral tumors, especially in older males [34]. In addition, such artefacts may also hinder the post-operative evaluation of bone grafts and implants [35,36]. In such cases, it is necessary to take MRI scans with altered settings, similar to the two affected patients in the present report. Finally, if symptoms of local Cu accumulation have been identified in the oral cavity, it is highly indicated that these outpatients are to be sent for detoxification therapy (e.g., with Cu-chelators or with drugs affecting Cu uptake/excretion) [37].

## 5. Conclusions

From the standpoint of oral health and oral health-related occupational exposures, musicians are a relevant but often neglected group. Herein, we described two cases of Cu-accumulation in the lips of professional brass players, which was identified via an MRI imaging scan, unrelated to an occupational disease workup. In summary, we have shown that these two patients showed a similarity in the artefact observed on MRI imaging and the discovered mucosal changes; nevertheless, a limitation of our study is, that only two patients were examined, thus, without the inclusion of additional patients, we cannot reliably conclude that the presented phenomenon points to a true occupational disease. However, as nowadays only coated mouthpieces are available almost exclusively, clinicians should be on the lookout for brass players involved in the profession for many years, who may have used uncoated mouthpieces for an extended period of time previously. On the other hand, brass players using the same coated (protective) mouthpieces for a longer time (as the longtime utility of coated mouthpieces is impacted by wear, and the protective layer may be partially disintegrated by the mechanical effects of the mouth and maceration due to saliva) and musicians simultaneously affected by Wilson’s disease should also be considered at risk. In patients affected by this phenomenon, diagnostics of oral cancer and prosthodontic procedures may be cumbersome, due to the detrimental impact on the utility of MRI imaging from artefact-formation and scattering. Targeted and systematic screening of brass players over a certain age limit would be helpful to identify such individuals, as a part of a more extended occupational medicine workup. 

## Figures and Tables

**Table 1:** Summary of patient characteristics.

	Patient 1	Patient 2
Sex	male	male
Age	63 years	61 years
Underlying conditions	none	glaucoma
Medication use	none	latanoprost
Known allergies	none	none
Tobacco and alcohol consumption	alcohol: very rarely tobacco: between 5–10 cigarettes/day	alcohol: very rarely tobacco: >25 cigarettes/day
Oral health status	fixed implants, metal-ceramic crowns	composite and amalgam fillings, metal-fused porcelain bridge in the upper right quadrant, chronic periodontitis, partially missing teeth
Type of mouthpiece	Stowasser	Schlagmüller U3
Instrument cleaning practices	infrequently, using Sidol	none reported

## Data Availability

All data generated during the study are presented in this paper.
